# ENU-Induced Mutagenesis in Grass Carp (*Ctenopharyngodon idellus*) by Treating Mature Sperm

**DOI:** 10.1371/journal.pone.0026475

**Published:** 2011-10-17

**Authors:** Xia-Yun Jiang, Cheng-Fei Sun, Quan-Gen Zhang, Shu-Ming Zou

**Affiliations:** Key Laboratory of Aquatic Genetic Resources and Utilization, Shanghai Ocean University, Shanghai, China; Auburn University, United States of America

## Abstract

*N*-ethyl-*N*-nitrosourea (ENU) mutagenesis is a useful approach for genetic improvement of plants, as well as for inducing functional mutants in animal models including mice and zebrafish. In the present study, mature sperm of grass carp (*Ctenopharyngodon idellus*) were treated with a range of ENU concentrations for 45 min, and then wild-type eggs were fertilized. The results indicated that the proportion of embryos with morphological abnormalities at segmentation stage or dead fry at hatching stage increased with increasing ENU dose up to 10 mM. Choosing a dose that was mutagenic, but provided adequate numbers of viable fry, an F1 population was generated from 1 mM ENU-treated sperm for screening purposes. The ENU-treated F1 population showed large variations in growth during the first year. A few bigger mutants with morphologically normal were generated, as compared to the controls. Analysis of DNA from 15 F1 ENU-treated individuals for mutations in partial coding regions of *igf-2a*, *igf-2b*, *mstn-1*, *mstn-2*, *fst-1*and *fst-2* loci revealed that most ENU-treated point mutations were GC to AT or AT to GC substitution, which led to nonsense, nonsynonymous and synonymous mutations. The average mutation rate at the examined loci was 0.41%. These results indicate that ENU treatment of mature sperm can efficiently induce point mutations in grass carp, which is a potentially useful approach for genetic improvement of these fish.

## Introduction

Genetic breeding of aquacultural fish species mainly depends on the discovery of natural mutants with high performance, and then improved strains can be produced through genetic selection, hybridization or marker-assisted breeding approaches [1], [2], [3]. To date, 73 improved breeds of aquacultural species have been produced in China, among which 39 strains were created by genetic selection, while the remainder were produced by hybridization [4]. All of these improved breeds were developed from existing natural mutants that carry desirable genes or traits [5], [6]. Thus, the acquisition of mutants with high performance is key for achieving breeding goals. However, the rate of natural spontaneous mutations in fish species is generally lower than 10^−6^, meaning that chemical mutagenesis is an efficient way to produce new mutants for future genetic improvement in aquacultural species [7].

Farmed fish are fertilized *in vitro* and display high reproductive abilities, which allows for chemical mutagenesis of the sperm, egg or embryo at various stages [7], [8], [9]. *N*-ethyl-*N*-nitrosourea (ENU) is a chemical mutagen that acts as an alkylating agent, transferring its ethyl group to nucleophilic nitrogen or oxygen sites on deoxyribonucleotides, leading to base mismatch during DNA replication [10], [11]. ENU treatment mainly induces single-base substitutions that resemble natural spontaneous mutations [9]. The mutation rate induced by ENU at specific loci ranges from 0.5×10^-3^ to 3.9×10^−3^ in mice and zebrafish, which is almost 10 times greater than mutation rates induced using any other means [12], [13]. In addition, mutations induced by ENU are unbiased meaning that all genes are mutated at random [14]. Induction of mutants using ENU has become an important method for examining the functional genome of model organisms including fruit flies, zebrafish, medaka, clawed frog and mice [15], [16]. Furthermore, though chemical mutagenesis has been used widely in genetic breeding of microorganisms and crops, the application of ENU in fish breeding has been reported only rarely [17], [18].

The grass carp, *Ctenopharyngodon idellus*, is an important herbivorous fish species for freshwater culture, with total annual production of 3.56 million tons in China alone [19]. The brood fish take at least five years to attain sexual maturity, and bodyweight usually exceeds 10 kg. Recently, Chinese investigators had made large effort in understanding genetic variations in natural and cultured populations using DNA markers and in developing genomic tools to facilitate breeding [20], [21], [22], [23], [24]. To date, however, breeds of grass carp with improved characteristics have not been produced by conventional mass selection due to its long life cycle. In this present study, we performed *in vitro* chemical mutagenesis of postmeiotic sperm, rather than *in vivo* spermatogonial treatment in grass carp, as this former method is easier in fish species with large bodyweights and it gives a high efficiency of mutation induction. After examining embryo development, first year growth and base changes of six growth-related genetic loci in an F1 population, ENU mutagenesis was shown to be efficient for inducing point mutations in the genome of the grass carp. In addition, large growth variations existed in the ENU-treated F1 population, which suggests that ENU mutagenesis may be a useful approach for future genetic breeding programs in grass carp.

## Materials and Methods

### Ethics Statement

This study was approved by the institutional review board or ethics committee of Shanghai Ocean University (Permit Number: 2009011). All experiments were conducted following guidelines approved by the Shanghai Ocean University Committee on the Use and Care of Animals.

### ENU working solution

All working solutions were freshly prepared following the methods described by Jin et al. [14]. Briefly, 0.1 g of ENU (Sigma) was dissolved in 8.5 mL of 10 mmol/L sodium acetate to give a 0.1 mol/L stock solution. Before use, the ENU stock solution was dissolved in modified Hanks solution to give 0.5, 1, 5 and 10 mmol/L working solutions. The modified Hanks solution contained 0.4 g KCl, 8 g NaCl 0.35 g NaHCO_3,_ 0.09 g NaH_2_PO_4_·7H_2_O, 0.1 g MgSO_4_·7H_2_O, 0.1 g MgCl_2_·6H_2_O, 0.06 g KH_2_PO_4_, 0.14 g CaCl_2_ and 1 g glucose in 1 L sterile deionized water.

### Experimental fish

The brood stock of grass carp was maintained at the Qingpu fish breeding facility of the Shanghai Ocean University. Well-developed 6-year-old broodfish were selected for use (four males and four females; mean body weight of 12 kg). The females were injected with 4 µg/kg luteinizing hormone-releasing hormone A2 (LHRH-A2, Ningbo Hormone Company, China), while the males received only half this dose. The broodfish were kept in a circular breeding pond (4 m in diameter) with flowing water stimulation. When broodfish displayed estrus and were ready to spawn, milts were manually stripped from males into a dry bowl and then these were treated immediately with ENU working solutions. Meanwhile, the female fish were kept in the breeding pond during the sperm treatment.

### ENU treatment, fertilization, hatching and cultivation

Four milliliters of each ENU working solution was mixed with 1 mL grass carp milts in 15-mL screw-cap centrifuge tubes and then incubated at room temperature for 45 min. Sperm activity was examined every 10 min under a light microscope. After ENU treatment, grass carp eggs were manually stripped into a dry bowl and mixed thoroughly with unwashed ENU-treated milts using a dry feather. Subsequently, water was added to activate the sperm for fertilization. Fertilized eggs were placed in Petri dishes (90 mm in diameter; ca. 200 eggs per dish) at room temperature. Water in the Petri dishes was replaced every 4 hrs with aerated pond water until the fry had hatched. The number of abnormal embryos at the segmentation stage and the survival rate at the hatching stage were determined. For each concentration of ENU, replicates were performed by fertilizing eggs from three different females with ENU-treated milts from three different males. Hatched fry were stocked into earth ponds and standard rearing procedures were continued during the first growing season. At the end of the year, fish were captured, labeled with passive integrated transponder (PIT) tags (Hongteng Barcode Corporation, Guangzhou) and weighed. Fin samples were collected and kept in 95% ethanol.

### Determination of F1 mutation sites

Total genomic DNA was isolated from fish fin clips (0.1 to 0.2 g) using a standard phenol-chloroform procedure detailed by Sambrook et al. [25]. Primers were derived from the published open reading frame sequences of *igf-2a*, *igf-2b*, *mstn-1*, *mstn-2*, *fst-1* and *fst-2* (Table 1), which were selected as growth-related genes [26], [27].

**Table 1:** Primers used in this study and their related amplification region in grass carp (*Ctenopharyngodon idellus*).

Locus	Primers 5′-3′	Size (bp)	Exon	Coding region (bp)
*igf-2a*	Forward: aacaggaggtcccaagaaa	263	4	389–651
	Reverse: tcacttgtggctaacgtagt			
*igf-2b*	Forward: tgtgaagtattccaaataga	214	4	393–606
	Reverse: tcatttgtgggatgtgttga			
*mstn-1*	Forward: atgcattttacgcaggtttt	396	1	1–396
	Reverse: gctctgtggccatggtcatg			
*mstn-2*	Forward: caagccatcacccatcttga	369	2	358–726
	Reverse: cagtccttcctctccagatt			
*fst-1*	Forward: aggccaagtcatgcgatgat	236	5	731–966
	Reverse: cttacagttgcaagatccta			
*fst-2*	Forward: agacgccaggtcctgtgaag	213	5	738–950
	Reverse: agttgcaggagcccgagtgc			

The PCR reactions (25 µL) contained 10 mmol/L Tris-HCl (pH 8.4), 20 mmol/L KCl, 10 mmol/L (NH_4_)_2_SO_4_, 1.5 mmol/L MgCl_2_, 0.1 mmol/L of each dNTP, 0.2 µmol/L of each primer, ca. 200 ng gDNA and 2 U pfu *Taq* DNA polymerase (Applied Biosystems). PCR thermal cycles comprised of one cycle of pre-denature (94°C for 5 min), followed by 35 cycles of amplification (94°C for 30 s, 50°C for 30 s, 72°C for 45 s), and a final extension step (72°C for 5 min). PCR products (3 µL) were analyzed by agarose gel electrophoresis, stained with ethidium bromide and photographed using a Bio-Rad gel image system.

Direct sequencing of the amplified regions of the six selected gene loci was performed in the parents, which can prevent noisy background from single nucleotide polymorphisms. PCR was carried out by forward primers (5′- GTTGTAACCTAGCTCTACTA -3′ for *igf-2a*, 5′- GCGAGATGTTTCCTCCACATC -3′ for *igf-2b*, 5′- CGGTGCGTGGTGAGGTTCATTTC -3′ for *mstn-1*, 5′- AGGATGAGGAACAAGGTAGC -3′ for *mstn-2*, 5′- GGCAACGATGGGATTGTTTAC -3′ for *fst-1* and 5′- TGCCATCTCCGAAGGGCCACTT -3′ for *fst-2*) and corresponding reverse primers in Table 1. PCR products were recovered from the gel and sequencing were performed on a 48 capillary 3730 DNA Analyzer (Applied Biosystems, Foster City, CA) using aforementioned forward primers following the methods described by Xu et al. [28]. To increase the probability of detecting mosaic mutation sites in the ENU-treated F1 progeny, which interferes with direct sequence analysis, clone sequencing of amplified regions was chosen to detect mutations at each gene locus. PCR products were recovered from the gel and cloned into the pMD19-T (TaKaRa) vector using *Escherichia coli DH5*α cells. Eight positive clones from each gene fragment were sequenced on a 48 capillary 3730 DNA Analyzer. Mutations were identified using software based on Polyphred version 6.0 Beta [29], which compares each trace to the parental reference sequence and identifies potential mutations. If a base change was seen in more than two eighths of the clones for each gene amplicon, it was categorized as a mutation. The following equation was used to calculate the mutation rate at each locus:



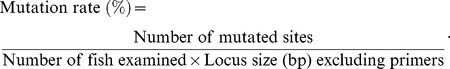



### Statistical analysis

Differences between groups were analyzed using one-way analysis of variance tests, followed by Fisher's post hoc test or unpaired *t*-tests. Significance was accepted at *p*<0.01.

## Results

### Effect of ENU treatment on embryo development of grass carp

**Figure 1:**
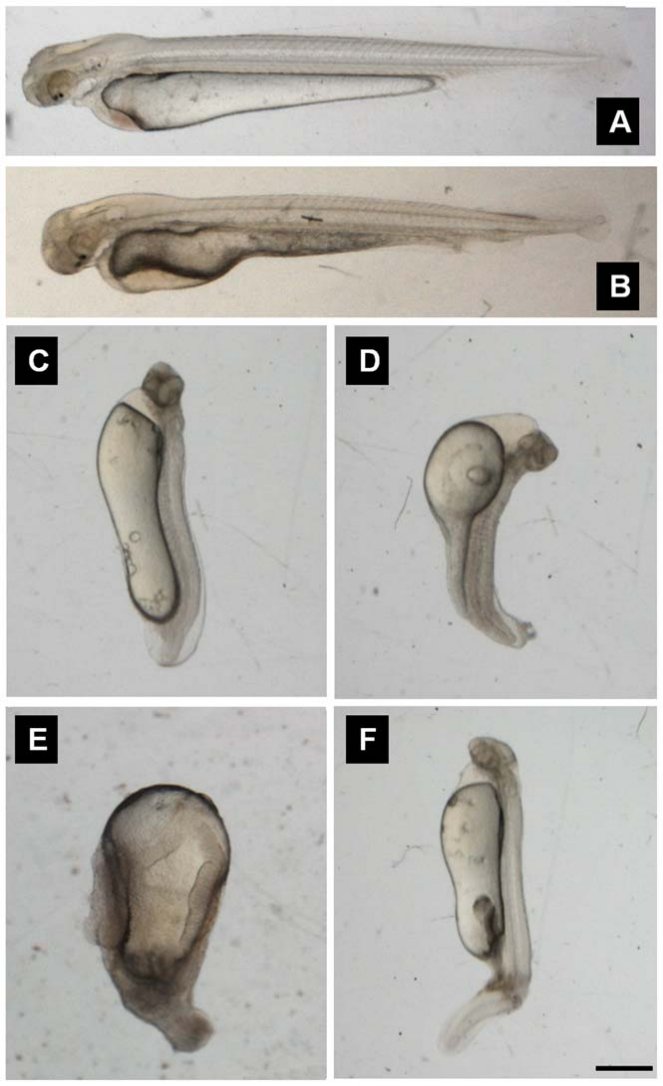
Morphological defects of ENU-treated F1 embryos at hatching stage. (A) Morphologically normal grass carp embryo. (B) Embryo with cardiac displacement. (C) Embryo with short spine and small head. (D) Embryo with short spine, small head, crooked tail and enlarged pericardial cavity. (E) Embryo without head. (F) Embryo with nervous system abnormalities, including a small head and crooked tail. Scale bar = 600 µm.

**Figure 2:**
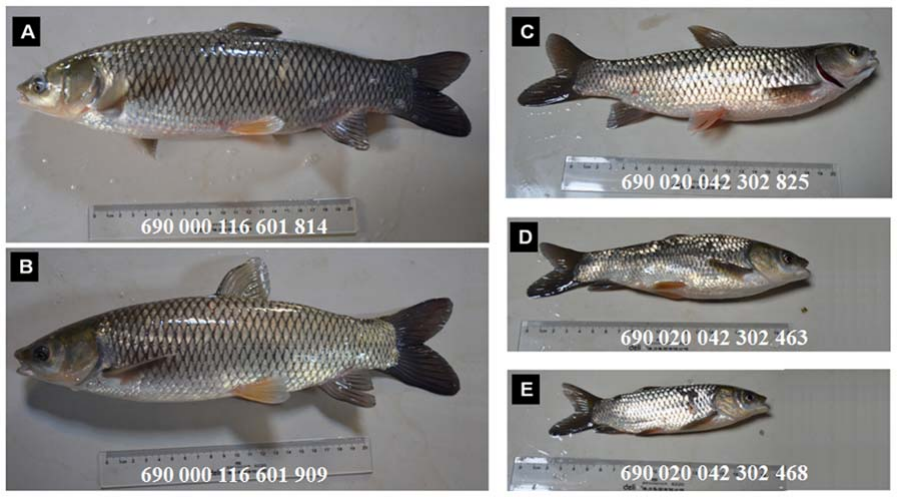
Morphology of ENU-treated F1 fish after eight months of rearing. (A, B) Morphologically normal grass carp individuals. (C, D, E) Grass carp individuals with various development retardation and body defects. The numbers in the photograph are the passive integrated transponder tag codes.

**Figure 3:**
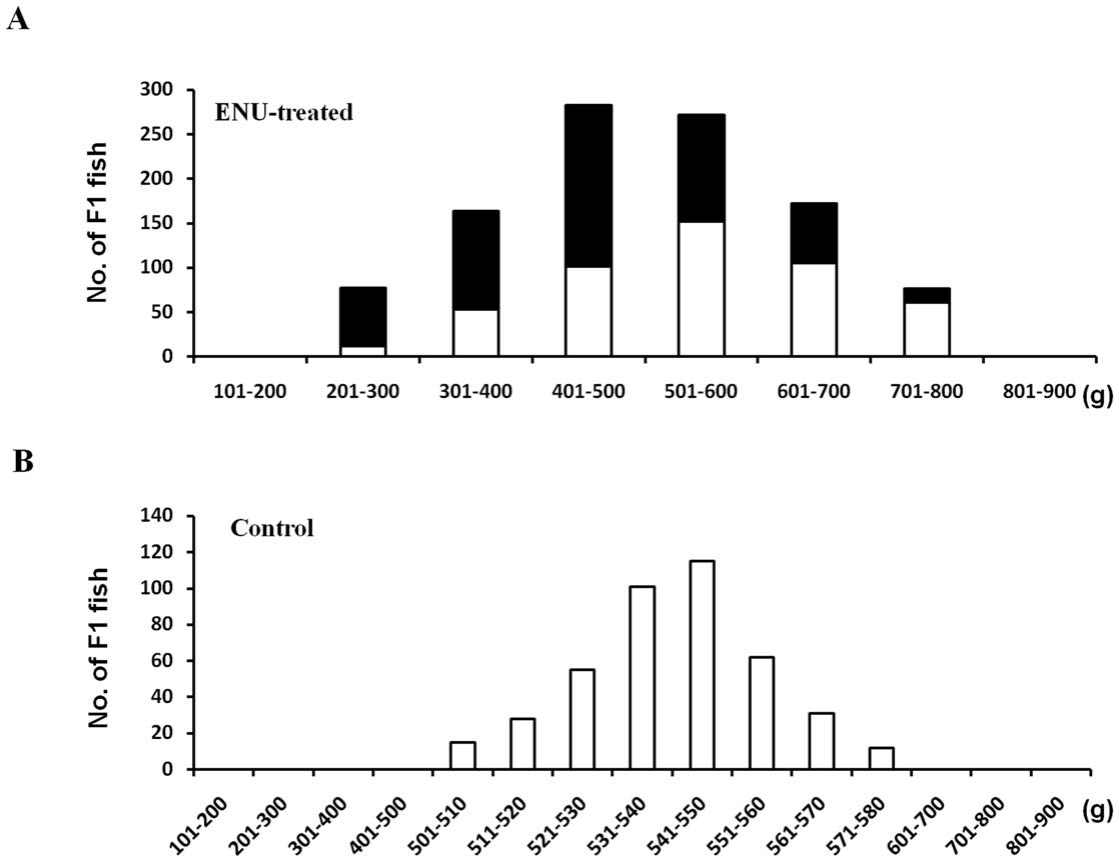
Growth distribution of ENU-treated F1 fish after eight months of rearing. (A) Distribution number of the ENU-treated F1 individuals with bodyweight from 201 to 800 g. (B) Distribution number of the F1 controls with bodyweight from 501 to 580 g. Black bar denotes morphologically abnormal individuals. White bar denotes morphologically normal individuals.

A range of ENU concentrations up to 10 mM was used to treat mature sperm for 45 min. These sperm were used to fertilize wild-type grass carp eggs, and the resulting embryos were scored for dominant effects and viability at the hatching stage. Treatment of mature sperm with 0.5, 1, 5, 10 mM ENU caused the formation of 16.1%, 38.7%, 66.9% and 91.3% abnormal embryos at the segmentation stage respectively, and these proportions were significantly (*P*<0.01) greater than the 2.8% seen in the control group (Table 2). Additionally, the survival rates at the hatching stage were 76.9%, 52.6%, 14.4% and 4.4% for the embryos created using sperm treated with 0.5, 1, 5, 10 mM ENU respectively, which were significantly (*P*<0.01) lower than the 93.4% survival seen in the control group (Table 2). Thus, the proportion of morphologically abnormal and dead fry during embryogenesis increased with increasing ENU concentration. Compared to a normal embryo (Fig 1A), defects seen in morphologically abnormal embryos included: (1) notochord abnormalities such as a shortened spine (Figs. 1C, 1D and 1E) or a crooked tail (Fig. 1F); (2) nervous system abnormalities such as a small head (Figs. 1C, 1D and 1F) or the absence of a head (Fig. 1E); (3) internal organ abnormalities such as heart displacement (Fig. 1B) or an enlarged pericardial cavity (Fig. 1D).

**Table 2:** Effects of different ENU concentrations on morphology of embryos and hatching success after treating mature sperm of grass carp.

ENU concentration (mM)	Crossing group	No. of embryos	Abnormality rate at segmentation stage (%)	Survival rate at hatching stage (%)
0.5	♀1×♂1	874	12.4	81.8
	♀2×♂2	653	14.6	76.2
	♀3×♂3	764	21.3	72.8
	Mean	764	16.1^a^	76.9^ a^
1	♀1×♂1	965	33.6	55.6
	♀2×♂2	654	42.3	52.4
	♀3×♂3	563	40.4	49.8
	Mean	727	38.7^ b^	52.6^ b^
5	♀1×♂1	567	60.6	19.6
	♀2×♂2	845	68.2	13.2
	♀3×♂3	265	71.9	10.4
	Mean	559	66.9^ c^	14.4^ c^
10	♀1×♂1	765	89.3	2.8
	♀2×♂2	565	88.2	9.3
	♀3×♂3	365	96.4	1.2
	Mean	565	91.3^ d^	4.4^ d^
0 (control)	♀1×♂1	745	2.8	93.6
	♀2×♂2	435	3.4	92.5
	♀3×♂3	542	2.3	94.2
	Mean	574	2.8^ e^	93.4^ e^

Different letters in the same column represent a significant difference between two groups (*P*<0.01).

### Growth variation in ENU-treated F1 individuals during the first year

As shown in Table 2, treatment of sperm with 1 mM ENU could produce adequate numbers of viable F1 individuals, which meanwhile displayed substantial dominant mutation effects. Thus, an F1 population was generated for screening purposes using sperm treated with this dose of ENU. The 1 mM ENU-treated F1 populations were derived from three pairs of parents as shown in Table 2. The ENU-treated F1 populations and untreated controls were reared separately in six earth ponds with the same conditions. After eight months of rearing, fish were captured, labeled with PIT, and then bodyweights were determined. Approximately 50% of the 1 mM ENU-treated F1 individuals were morphologically normal (Fig. 2A and 2B), but the others displayed various extents of development retardation and body defects (Figs. 2C, 2D and 2E). As shown in Fig. 3A, the bodyweights of the ENU-treated F1 individuals ranged from 204.5 to 756.6 g, with a mean bodyweight of 437.1 ±276.2 g (± one standard deviation) (Table 3). The bodyweights of F1 control fish ranged from 504.2 to 576.4 g, with a mean bodyweight of 548.7 ±42.4 g (Table 3, Fig. 3B). Although the mean bodyweight of the ENU-treated F1 population was only 80% of the control fish, the standard deviation of bodyweight was 6.5-fold greater in the ENU-treated F1 population than in the control population. In the ENU-treated F1 population, more than 85% (478/560) of morphologically abnormal individuals were of lower (<600g) bodyweight, while a portion of 62% (166/484) progenies with bodyweight bigger than 600g were morphologically normal (Fig. 3). These morphologically normal mutants with high growth rates in the ENU-treated F1 populations may be useful in future breeding.

**Table 3:** Growth of ENU-treated F1 fish after eight months of rearing.

Crossing group	ENU concentration (mM)	No. of F1 fish	Body weight range (g)	Body weight (g)
♀1×♂1	1	304	218.3–756.6	431.2±270.1
	0 (control)	102	511.7–576.4	555.8±44.7
♀2×♂2	1	322	212.1–744.2	452.9±292.4
	0 (control)	128	504.2–565.5	538.5±40.3
♀3×♂3	1	418	204.5–750.6	427.2±266.1
	0 (control)	189	519.3–559.8	551.8±42.2
Mean	1	—	—	437.1±276.2^ a^
	0 (control)	—	—	548.7±42.4^ b^

Different letters in the same column represent a significant difference between two groups (*P*<0.01).

### Detection of mutation sites and frequencies in the ENU-treated F1 population

To determine the mutation sites and frequencies in the F1 population derived from the 1 mM ENU-treated sperm, genomic DNA was isolated from 15 ENU-treated F1 fish and five control fish, which were derived from a single pair of parents. Sequences of six gene loci (*igf-2a*, *igf-2b, mstn-1, mstn-2, fst-1* and *fst-2*) were analyzed. As shown in Table 4, the amplified fragments (excluding the primers) were 224 bp, 174 bp, 356 bp, 329 bp, 196 bp and 173 bp for *igf-2a*, *igf-2b, mstn-1, mstn-2, fst-1* and *fst-2* respectively. Among the 15 ENU-treated individuals, there were 15, 10, 18, 21, 12 and 12 point mutations at these loci respectively, while the mutation rates were 0.45%, 0.38%, 0.34%, 0.43%, 0.41% and 0.46% respectively (Table 4). No mutations were identified at these gene loci in the F1 control individuals.

**Table 4:** Mutation rates in partial coding regions at six selected gene loci.

Locus	Size (bp) excluding primers	Group	No. of fish examined	No. of clones sequenced/fish	No. of mutated sites	Mutation rate (%)
*igf-2a*	224	ENU-treated F1	15	8	15	0.45
		F1 control	5	8	0	0
*igf-2b*	174	ENU-treated F1	15	8	10	0.38
		F1 control	5	8	0	0
*mstn-1*	356	ENU-treated F1	15	8	18	0.34
		F1 control	5	8	0	0
*mstn-2*	329	ENU-treated F1	15	8	21	0.43
		F1 control	5	8	0	0
*fst-1*	196	ENU-treated F1	15	8	12	0.41
		F1 control	5	8	0	0
*fst-2*	173	ENU-treated F1	15	8	12	0.46
		F1 control	5	8	0	0
Average	242	ENU-treated F1	15	8	15	0.41
		F1 control	5	8	0	0

Mutation rate (%) = Number of mutated sites/Number of fish examined×Locus size (bp) excluding primers.

**Figure 4:**
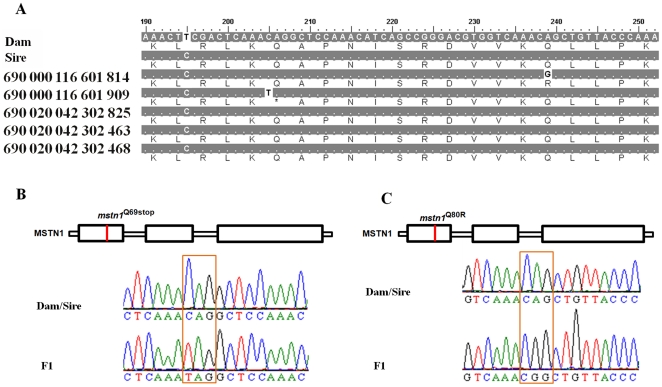
Mutation sites at the *mstn1* locus. (A) Partial *mstn1* sequence alignment of five ENU-treated F1 juvenile fish with sequences from their parents. The numbers by the sequences correspond to the passive integrated transponder tags as shown in Figure 2. (B) Location of the MSTN1^Q69stop^ mutation and sequencing that revealed a C to T point mutation. (C) Location of the MSTN1^Q80R^ mutation and sequencing that revealed an A to G point mutation. Dam and sire denote to female and male, respectively.

**Figure 5:**
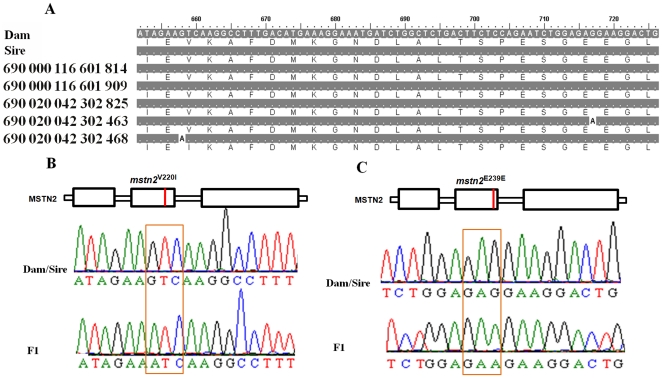
Mutation sites at the *mstn2* locus. (A) Partial *mstn2* sequence alignment of five ENU-treated F1 juvenile fish with sequences from their parents. The numbers by the sequences correspond to the passive integrated transponder tags as shown in Figure 2. (B) Location of the MSTN2^V220I^ mutation and sequencing that revealed a G to A point mutation. (C) Location of the MSTN2^E239E^ mutation and sequencing that revealed a G to A point mutation. Dam and sire denote to female and male, respectively.

Among the nucleotide substitutions seen at the six selected gene loci in ENU-treated F1 individuals, 52% (46/88) were GC to AT transitions, 35% (31/88) were AT to GC transitions, 9% (8/88) were AT to TA transversions, two was an AT to CG transversion and one was a GC to TA transversion (data not shown). These substitutions led to nonsynonymous changes in approximately 66% (58/88) of cases, of which approximately 64% (37/58) were missense changes, while the remainder gave nonsense mutations. As shown in Fig. 4, an individual with PIT 690,000,116,601,909 (see Fig. 2B) had a C to T point mutation at nucleotide 205 within the 190 bp to 252 bp region of *mstn1*, which resulted in the Gln at position 69 being substituted with a stop codon (Fig. 4A and 4B). The individual with PIT 690,000,116,601,814 (see Fig. 2A) had an A to G mutation at nucleotide 239, which resulted in a Gln to Arg substitution at position 80 (Figs. 4A and 4C). Additionally, within the coding region of 652 bp to 726 bp of *mstn2* an individual with PIT 690,020,042,302,468 (see Fig. 2E) showed a G to A mutation at nucleotide 658, which resulted in a Val to Ile substitution at position 220 (Fig. 5A and 5B). A further individual with PIT 690,020,042,302,463 (see Fig. 2D) showed a G to A change at nucleotide 717, which resulted in a synonymous substitution (Figs. 5A and 5C).

## Discussion

Compared to the low natural spontaneous mutation frequency in fish, chemical mutagenesis can be useful for increasing genetic mutations. Highly efficient mutagens can induce profound changes in genetic material and produce mutants with desirable traits. Studies with ENU in the cyprinidae model species zebrafish have obtained mutations in hundreds of genes that are required for embryonic viability [30], [31]. ENU-induced mutants for targeted genes have also been reported for medaka [32], [33]. Although ENU mutagenesis is a potential method for genetic breeding in commercial fish species, the successful generation of improved carp strains by ENU mutagenesis combined with gynogenesis has only been reported by Kirpichnikov [18]. Recently, Han et al. [34] reported ENU mutagenesis studies in the gibel carp (*Carassius autatus gibeblio* Bloch) by *in vivo* spermatogonial treatment. In this present study, application of ENU mutagenesis in the grass carp by treating mature spermatozoa was explored. The 1 mM ENU-treated F1 population of the grass carp showed greater variations in bodyweight during the first year of growth compared with the control group. Although the mean bodyweight of the ENU-treated F1 population decreased to 80% of the controls, the standard deviation of bodyweight in the ENU-treated group was 6.5-fold greater than the controls. A few morphologically normal, but bigger individuals in theENU-mutated F1 population may provide a useful resource for further functional gene identification studies and genetic improving programs of grass carp.

ENU mutagen treatments can influence the fertilization abilities of sperm [15]. By discarding embryos that displayed defects prior to neurulation stages, it is possible to efficiently screen for later phenotypes in patterning, organogenesis and differentiation. In the present study, the proportion of morphologically abnormal or dead hatching stage embryos increased with increasing dose of ENU. ENU mutagenesis produced various dominant mutations and/or synthetic lethal mutations, in which genetic material has been modified. The dominant phenotypes caused by ENU treatment in grass carp led to the disruption of specific embryonic development processes, such as notochord patterning and nervous system and internal organ development. Nevertheless, both forward and reverse genetic screens will be required to confirm whether the mutations are responsible for the dominant phenotypes observed in the ENU-treated progeny [35]. Once a mutation is identified, homozygous mutants with high performance can ultimately be generated in the F3 generation [15].

In the present study, mutations were induced *in vitro* by ENU treatment of postmeiotic sperm. The initial mutation, usually occurred with an ethylation of a base on one DNA strand, leads to mosaic offspring in the next generation that interferes with direct sequence analysis in the F1 generation. To detect mutations in the partial coding regions of *igf-2a, igf-2b, mstn-1, mstn-2, fst-1* and *fst-2,* clone sequencing of amplified regions was performed. The results showed that these loci displayed mainly GC to AT or AT to GC substitutions, resulting in nonsynonymous nonsense or missense mutations (66%), as well as synonymous mutations. The induced point mutations in the grass carp were similar to those seen in mammalian cells [9], [36], where most ENU-induced mutations are GC to AT transitions, and to a lesser extent AT to GC transitions, although all types of transitions and transversions have been documented after exposure to ENU. Interestingly, neither deletions nor translocations were identified in this present study, however such mutations have been induced in zebrafish by postmeiotic ENU treatment of male germ cells [35]. It is possible that multigene deletions are induced by the protocol used in this present study, and these may result in a higher frequency of early lethal phenotypes, which were discarded. On the other hand, it has also been proposed that subtle differences in mutagenesis conditions may result in significant differences in the kinds of lesions produced [15].

Mutation rates caused by ENU range from 0.5×10^−3^ to 3.9×10^−3^ at specific loci when *in vivo* spermatogonial treatment has been used in mice and zebrafish [12], [13]. Moreover, ENU mutagenesis in medaka after *in vivo* spermatogonial treatment induces mutation rates of 1×10^−3^ to 1.9×10^−3^ [8], [33]. Genetic and molecular tests have shown that postmeiotic ENU treatment can induce point mutations [37], but the range of mutations induced has not been analyzed extensively. In this present study, the average point mutation rate was 4.1×10^−3^ at six selected gene loci in grass carp after *in vitro* chemical mutagenesis of postmeiotic sperm. These results indicate that treatment of postmeiotic gametes with ENU induces point mutations at a higher rate than premeiotic regimens, suggesting that postmeiotic mutagenesis protocols could be useful in genetic screening strategies. Postmeiotic mutagenesis has been reported to produce a 10-fold increase in the frequency of induced mutations in specific-locus tests in zebrafish [35].

In summary, the ENU-treatment of mature sperm with different doses can markedly generate dominant effects on embryo development of grass carp. The ENU-treated F1 populations demonstrated large variations in bodyweight during the first year. Some bigger mutants with morphologically normal were produced in the ENU-treated F1 progeny, which may be useful for genetic breeding in future. Our further sequence data showed that the postmeiotic ENU treatment can efficiently induce point mutations. Most of these point mutations were GC to AT or AT to GC substitutions that led to nonsense, nonsynonymous and synonymous mutations. The classical three-generation of breeding or two-generation gynogenetic screen may be used to confirm mutants that carry desirable genes or traits for breeding in future.
